# Uncertainty, Search Engine Data, and Stock Market Returns During a Pandemic

**DOI:** 10.3389/fpubh.2022.884324

**Published:** 2022-04-06

**Authors:** Sheng Xu, Jing Zhang, Rui Shen

**Affiliations:** ^1^School of Economics, Zhejiang University of Technology, Hangzhou, China; ^2^School of Economics and Management, Southwest Jiaotong University, Chengdu, China

**Keywords:** COVID-19, uncertainty, search engine data, stock returns, pandemic

## Abstract

In recent years, a series of uncertain events, including the spread of COVID-19, has affected the Chinese stock market. When people face uncertainty, they often turn to internet search engines to obtain more information to support their investment decisions. This paper uses the uncertainty index, investor sentiment reflected by search engine data, and Chinese stock return data during the pandemic to examine the relationships among the three. Using daily data from March 2, 2020, to March 2, 2021, our empirical findings reveal that stock returns during a pandemic lead to an increase in investor retrieval of search engine data and that uncertainty affects stock returns during a pandemic. However, the reverse is not true. Therefore, in the face of an uncertainty such as market volatility caused by the spread of the pandemic, the active release of favorable information by regulators can help guide investor sentiment, prevent sharp stock market volatility, and improve the effectiveness of policy governance.

## Introduction

In recent years, the U.S.–China trade war, the spread of COVID-19, and, most recently, the war between Russia and Ukraine, have heightened uncertainty in the global economy. These events not only caused huge losses to the global economy but also had a significant impact on the Chinese stock market, resulting in large fluctuations in stock indices. Without strong regulation, this uncertainty fuels speculation and affects the stability of financial markets and even the broader economy. This uncertainty not only affects investor confidence and increases investors' holdings of short-term liquid assets such as cash, it can also cause investors to have difficulty predicting the future. Additionally, consumers and investors are also reluctant to make spending and investment decisions when they sense a high level of uncertainty in the economy. Economic contractions cause recessions to become more pronounced when businesses delay investment. The cost of financing for investors is also related to uncertainty: High levels of uncertainty lower asset prices and drive up financing costs, leading to reduced investment and a slowdown in the economy. Furthermore, these uncertainties that result from politics, war, and the pandemic are also among the main obstacles to a global recovery from the downturn. Therefore, it is particularly important to study the impact of uncertainty on economic or investment decisions.

A significant challenge for this field of research is finding appropriate metrics to measure uncertainty. Uncertainty is a broad concept that involves not only macro phenomena such as growth in the gross domestic product but also micro phenomena such as corporate growth rates as well as other factors such as elections, wars, pandemics, and the impacts of climate change. Given these challenges, researchers employ a number of different methods to measure uncertainty. Some scholars use the volatility of key economic and financial variables. Another method is the World Uncertainty Index constructed by the International Monetary Fund. Moreover, Baker et al. (1) constructed economic and policy uncertainty indices based on text searches. Scholars have explored the impact of uncertainty on various economic indicators and financial markets using a number of indicators, including aspects such as democracy, unemployment, real estate returns, exchange rates, demand for money, business investment, and the stock market.

However, these uncertainty indices have certain limitations. Most are based on developed economies, creating a knowledge gap regarding uncertainty as it relates to developing countries. Even when uncertainty indicators are available, they are usually based on low-frequency data in units of years or months. This time lag not only offers limited assistance in making investment decisions, it also can be misleading at times, limiting the usefulness of low-frequency data in investment forecasting. In reality, more and more investors are relying on the information provided by search engines to help their decision-making. These high-frequency data provided in nearly real time can help to make up for the lack of self-information, thus providing a useful reference for decision-making. However, if the channels for investors to obtain information sources are too narrow, the information exchange among investors can easily lead to the herd effect, which will have an impact on investment behavior, stock prices, and even the overall financial market. Therefore, it is of great practical significance to study uncertainty and search engine data and their impact on China's stock returns during the current pandemic.

Based on these challenges, this paper constructs new uncertainty indicators and investor sentiment indicators based on the query data of Baidu, the largest search engine in China, to analyze uncertainty, investor sentiment, and their impact on stock returns during the pandemic. This paper seeks to provide new insights in this regard.

## Literature Review

In recent years, some scholars have used search engine data such as Google Trends, Google Analytics, and Baidu as new data sources for tourism demand, visitor number forecasting, and travel route selection. Yang et al. (2) used Google Trends and Baidu search data to predict the number of tourists in Hainan Province. The results of that study indicate that the use of Google Trends and Baidu search data can improve the predictive ability of a model. However, the predictive power of the Baidu Index (A tool to track online searches of specific keywords in China) is better than that of Google search data, mainly due to the fact that more Chinese users use the Baidu search engine to retrieve data and provide information. Sun et al. (3) proposed a new framework integrating machine learning and internet search indexing to predict the number of tourists at popular destinations in China and compared the predictive performance of the framework with search results generated by Google and Baidu. Huang et al. (4) used Baidu to predict the flow of tourists at the Forbidden City.

Other studies have examined the application of the Baidu Index in the financial sector. Zhang et al. (5–7) applied the Baidu Index to the prediction of Shanghai Stock Exchange 50 Index and COVID-19. Shen et al. (13) studied whether the Baidu Index can be used to predict Chinese stock returns, arguing that the Baidu Index search frequency can predict the price changes the next day. The stock price goes up when the retail investor pays less attention to the stock, and the stock price falls when the retail investor pays a significant amount of attention to the stock. Lang et al. (8), however, believed that information from Internet forums was superior to search frequency in predicting stock market volatility. Liu et al. (9) used Google Trends and the Baidu Index to analyze the impact of disaster events on company stock prices. Research indicates that the onset of a disaster can temporarily increase stock volatility and can have a significant negative impact on prices over an extended period of time.

It is also common to use the Baidu Index for pandemic prediction. Li et al. (15) used the Baidu search engine to monitor the AIDS global epidemic, and their study concluded that Baidu search query data are a useful indicator for reliable monitoring and prediction of the HIV and AIDS epidemic in China. Zhao et al. (10) argued that the Baidu Index could enhance traditional surveillance systems to monitor and predict the prevalence of brucellosis in China. Fang et al. (14) also used the Baidu Index to predict Huawei's mobile phone sales. Wu et al. (11, 12, 16) examined the impact of uncertainty on cryptocurrencies, medical products and labor markets.

## Data Sources and Statistical Description

Pandemic-era data on Walvax Biotechnology (300142.SZ) come from the Chinese Shenzhen Stock Exchange and Accounting Research database. The company is a high-technology biopharmaceutical enterprise specializing in research and development, production, and sales of biotechnology drugs such as human vaccines. We use the following formula to calculate stock returns:


$$Return_{i,t}\;=\;\frac{\left(Closing\;price_{i,t}-Closing\;price_{i,t\;-1}\right)}{Closing\;price_{i,t\;-1}}$$

with *Closing price_i,t_* representing the closing price of stock *i* on day *t*. All stock return figures are multiplied by 100. The search keywords like “Covid-19 latest news” come from the Baidu Index (the largest search engine in China), which is represented by search to indicate investor sentiment and judgment on future trends. The uncertainty index is constructed by the search keywords like “Wuhan epidemic”, and “coronavirus vaccine” related to uncertainty, which is represented by *Uncertainty*. The interval studied in this paper is from March 2, 2020, to March 2, 2021.

The data in Table 1 indicate that the maximum return of stocks during this period of the pandemic is 12.42287, and the minimum is −19.9956. The search data were between 9.22 and 11.4, and the uncertainty index ranged from 14.32 to 20.03. There are 244 observations for each variable.

**Table 1:** Statistical description of the main variables.

	**Mean**	**Maximum**	**Minimum**	**Std. dev**.	**Observations**
Return	0.265005	12.42287	−19.9956	3.808179	244
Search	10.14416	11.43895	9.220291	0.436147	244
Uncertainty	16.49937	20.02864	14.31843	0.983669	244

## Empirical Research

First, we examined the stationarity of the data through the augmented Dickey–Fuller (ADF) test. When using the ADF for the unit root test, the choice of the model form is more important. By observing the trend of the time series, we judged whether the package intercept term or trend term should be included in the model, and then determined the final form of the model by examining the coefficients of variables such as the intercept term, the time trend term, and the unit root. The determination of the lag order is based mainly on the Schwarz information criterion.

The unit root test in Table 2 indicates that the original time series of the variables *Search* and *Uncertainty* are not stationary, but the first-order difference series of the three variables are stationary. Therefore, we can perform the Granger causality test on the first difference of the above variables to examine the influence relationship among the three variables. Because the selection of the lag item has a significant influence on the results of the Granger causality test, we selected various lag orders to test the Granger causality among the three variables. Finally, we choose the lag item as 1, and the empirical research results indicate (see Table 3) that, under the condition of 10% significance, the influence relationship among variables is more of a one-way influence. Stock returns during the pandemic are the Granger reason for the Baidu Index, but not the reverse. That is to say, when the stock market fluctuates, investors usually turn to the Baidu search engine to retrieve relevant stock-related information. However, the retrieval of information did not lead to fluctuations in the stock prices during the period of pandemic under study. Because of the low quality of data from search engines, this information has limited impact on investment decisions, but it is clear that uncertainty is what is weighing on the returns during the period studied. Faced with uncertainty, investors often resort to recouping their investment or to a more conservative investment strategy. This inevitably led to changes in the returns of the stocks during the first year of the pandemic. However, the reverse is not true: There is no causal relationship between uncertainty and the Baidu Index.

**Table 2:** ADF test for the main variables.

	**ADF statistic**	**The cutoff value at the 1% significance level**	**Schwarz Criterion**	**Stationarity**
RETURN	−16.02335	−2.574513	5.536233	Stationary
ΔRETURN	−11.57494	−2.574756	5.739053	Stationary
SEARCH	−3.086717	−3.996271	−0.627749	Non-stationary
ΔSEARCH	−14.91810	−2.574553	−0.629616	Stationary
Uncertainty	−3.442558	−3.457173	1.125236	Non-stationary
ΔUncertainty	−16.27744	−2.574553	1.142875	Stationary

**Table 3:** Granger test results.

**Null Hypothesis:**	**Obs**	**F-Statistic**	**Prob**.
SEARCH does not Granger Cause RETURN	242	0.47462	0.4915
RETURN does not Granger Cause SEARCH	3.42235	0.0656	
UNCERTAINTY does not Granger Cause RETURN	242	2.97032	0.0861
RETURN does not Granger Cause UNCERTAINTY	0.27909	0.5978	
UNCERTAINTY does not Granger Cause SEARCH	242	0.33030	0.5660
SEARCH does not Granger Cause UNCERTAINTY	1.39308	0.2391	

**Figure 1:**
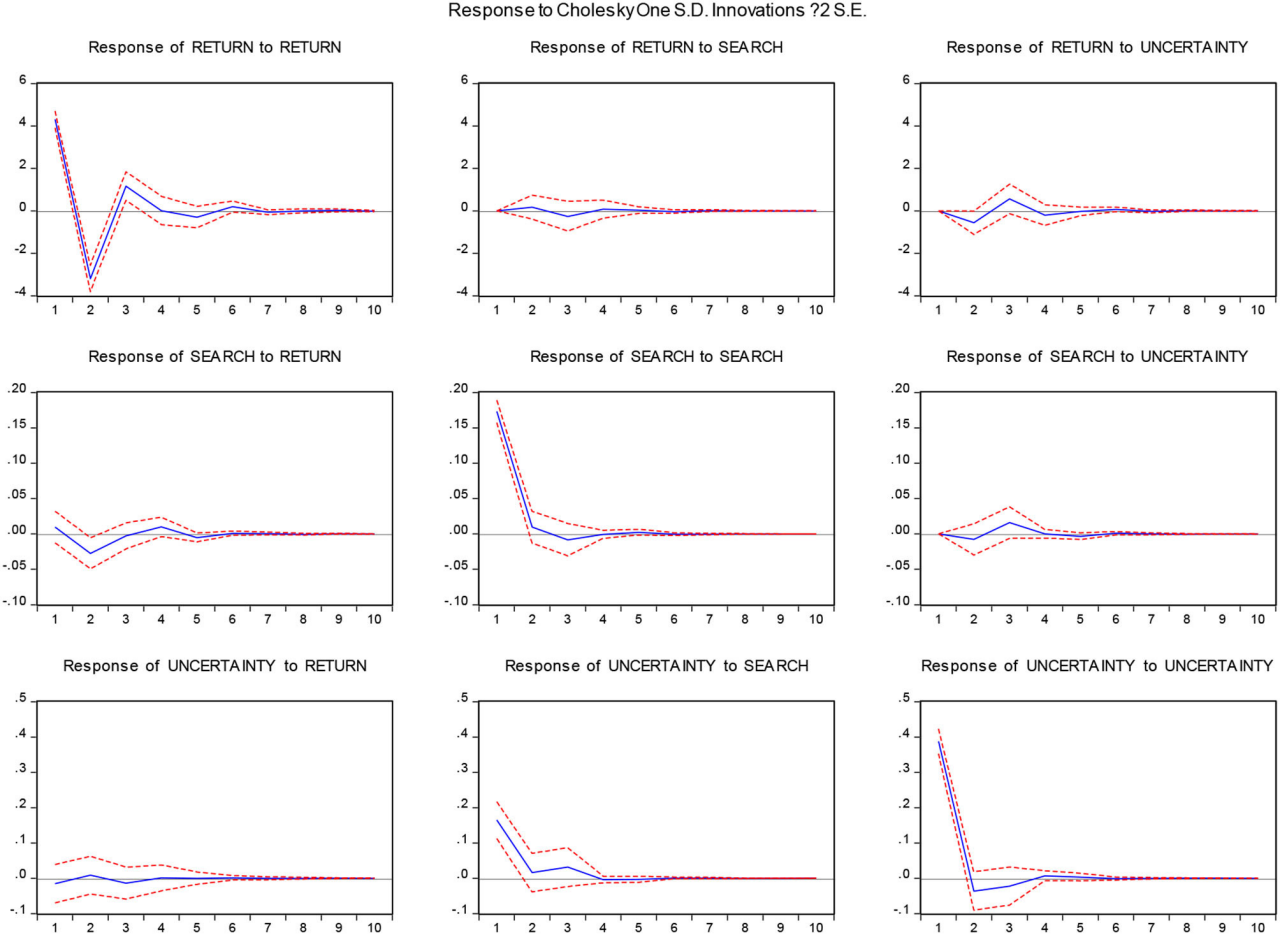
Impulse response function among main variables.

We investigated the cointegration relationships among variables and found no cointegration relationship between the original time series, so we took the first-order difference form to build the vector autoregression model. The calculation results of the impulse response function in Figure 1 reveal that the response of the stocks to the Baidu Index shock during the pandemic period is relatively small. However, uncertainty has a larger impact on the return of stocks during the period studied, and most of this impact is negative. Therefore, stock returns during the pandemic have a significant impact on Baidu searches. This is consistent with the conclusions of the Granger causality test. The response to uncertainty regarding stock returns during the pandemic has been less clear, but the search index adds to the uncertainty.

## Main Conclusions and Discussions

In the face of external economic and policy uncertainty, it is of important theoretical significance and policy value to examine the impact of investor sentiment on financial markets. Using policy uncertainty indicators and investor sentiment as represented by internet search engines, this paper enriches research in this area by examining policy uncertainty, investor sentiment, and their im pact on stock returns during the first year of the pandemic. This paper reveals the results of the study using data from March 2, 2020, to March 2, 2021, with respect to a series of policy uncertainties related to the pandemic that affect the stock returns. There is a cointegration relationship between policy uncertainty, investor sentiment, and stock returns during the pandemic. Fluctuations in stock returns during this period can lead to an increase in Baidu searches but not the reverse. Uncertainty is what is weighing on the stock returns, and the shock effect of uncertainty on these returns has lasted for a long time.

Based on these research results, we believe that, in the face of economic and policy uncertainties such as the impact of a pandemic, investor behavior is likely to resemble a herd effect. Therefore, real-time monitoring of investor sentiment in search engines, stock bars, and other investor gathering areas as well as active guidance of investors' public opinion orientation have an irreplaceable role in improving the effect of policy governance. In particular, the use of high-frequency data such as that provided by search indices can effectively alleviate the drawbacks of traditional low-frequency data that lag in policy responses when new situations and problems arise. Additionally, when companies or investors face external economic or policy uncertainty, collecting high-frequency real-time data from search engines or stock bars can provide valuable information for decision-making, thereby effectively making up for the lack of information. As a result, asymmetry effectively reduces investment risk in times of uncertainty.

## Data Availability Statement

Publicly available datasets were analyzed in this study. This data can be found here: https://index.baidu.com.

## Author Contributions

SX: data curation and literature. JZ: writing the original draft. RS: methodology and writing. All authors contributed to the article and approved the submitted version.

## Funding

Thanks for the research support by the Ministry of Education of China (19YJA790072).

## Conflict of Interest

The authors declare that the research was conducted in the absence of any commercial or financial relationships that could be construed as a potential conflict of interest.

## Publisher's Note

All claims expressed in this article are solely those of the authors and do not necessarily represent those of their affiliated organizations, or those of the publisher, the editors and the reviewers. Any product that may be evaluated in this article, or claim that may be made by its manufacturer, is not guaranteed or endorsed by the publisher.
